# A randomized, controlled clinical trial of autologous stromal vascular fraction cells transplantation to promote mechanical stretch-induced skin regeneration

**DOI:** 10.1186/s13287-021-02318-5

**Published:** 2021-04-15

**Authors:** Poh-Ching Tan, Pei-Chuan Chao, Chen Cheng, Chu-Hsin Chen, Ru-Lin Huang, Shuang-Bai Zhou, Yun Xie, Qing-Feng Li

**Affiliations:** Department of Plastic & Reconstructive Surgery, Shanghai Ninth People’s Hospital, Shanghai Jiao Tong University School of Medicine, 639 Zhizhaoju Road, Shanghai, 200011 People’s Republic of China

**Keywords:** Stem cell, Stromal vascular fraction (SVF), Mechanical stretch, Skin regeneration, Skin expansion

## Abstract

**Background:**

The regeneration response of the skin to mechanical stretching in vivo has been explored in reconstructive surgery to repair large-scale deformities. The ability of the skin to regenerate limits the reconstructive outcome. Here, we propose an approach in which autologous stromal vascular fraction (SVF) cells and mechanical stretching are combined to overcome this limitation and promote skin regeneration.

**Methods:**

This randomized, blinded, placebo-controlled clinical trial screened 22 participants undergoing tissue expansion with exhausted regeneration. Twenty eligible participants received intradermal injections of the SVF or placebo treatments. Follow-ups were conducted at 4, 8, and 12 weeks to assess efficacy and at 2 years to assess safety. The primary endpoint was the expanded skin thickness at 12 weeks. The secondary endpoints included skin thickness at 4 and 8 weeks, the expansion index (EI), and the skin texture score at 12 weeks.

**Results:**

The skin thickness of the SVF group was significantly higher than that of the control group at both 8 weeks (mean difference 0.78 [95% CI − 1.43 to − 0.11]; *p* = 0.018) and 12 weeks (0.65 [95% CI − 1.30 to − 0.01]; *p* = 0.046). In the SVF group, the increase in skin thickness was significant at 4 weeks (0.49 [95% CI − 0.80 to − 0.06]; *p* = 0.010) to 8 weeks (0.45 [95% CI − 0.92 to 0.02]; *p* = 0.026) and maintained after 12 weeks, whereas that in the control group was reduced after 8 weeks (0.42 [95% CI − 0.07 to 0.91]; *p* = 0.037). The SVF group showed greater EI increases than the control group (0.50 [95% CI − 0.00 to 0.99]; *p* = 0.047). The skin texture scores in the SVF group were greater than those in the control group at 12 weeks. Histologically, SVF-treated expanded skin showed more proliferating cells and blood vessels, and the extracellular matrix volume increased. No severe adverse events occurred.

**Conclusions:**

Transplantation of SVF cells can expedite the potency of mechanical stretch-induced skin regeneration and provide clinical reconstruction with plentiful tissue.

**Trial registration:**

This trial was registered with the Chinese Clinical Trial Registry, ChiCTR2000039317 (registered 23 October 2020—retrospectively registered).

## Background

Repairing large skin defects remains a major clinical challenge due to a lack of suitable tissue. Soft tissue expansion, which induces in vivo skin regeneration via mechanical stretching, is a reliable method for massive skin deficiency reconstruction [1]. Continuous mechanical stretch induced by inflating subcutaneous silicone expanders stimulates cell proliferation and extracellular matrix (ECM) synthesis [2] and creates vascularized skin tissue with an integrated structure and a well-matched texture [3].

However, in patients with massive skin defects, the amount of skin needed often exceeds the amount that can be provide via skin expansion. The growth capacity of skin can only support expansion to two to three times that of the original area. Overexpansion leads to skin thinning, ischemia, and possibly even necrosis [4–6] and severely limits reconstructive outcomes. Furthermore, the skin expansion routine typically takes more than 6 months with an increased incidence of adverse events [7, 8]. Therefore, new methods for promoting skin regeneration and accelerating inflation are needed to challenge the limitations of soft-tissue reconstruction.

Over the last decade, the excitement about stem cell research has been unprecedented [9, 10]. Adult stem cells and their derivatives are emerging as a promising therapeutic agent for tissue repair and regeneration [11–17]. In our preclinical studies, we reported that mesenchymal stem cells can effectively promote mechanical stretch-induced skin regeneration by differentiation and growth factor secretion [18–23]. Adipose tissue is a large and easily accessed depot of stem/progenitor cells. Stromal vascular fractions (SVF), which are separated from adipose tissue by enzymatic digestion or mechanical manipulation [24], represent a heterogeneous cellular population enriched with stem cells and progenitors [25–28]. Recent studies reported that SVF participates in ectoderm and endoderm tissue repair and regeneration, including the modulation of inflammation, cell proliferation, and ECM synthesis with the formation of new blood vessels and granulation [29–32]. Our preclinical study showed that intradermal transplantation of SVF promoted skin expansion [20, 21]. In our study, we proposed that SVF treatment promotes skin regeneration under mechanical stretching and optimized skin expansion limitations.

In this single-blinded, randomized, controlled clinical trial, we aimed to evaluate the safety and efficacy of intradermal transplantation of autologous SVF in promoting mechanical stretch-induced skin regeneration.

### Method study design

The study was a parallel, single-blinded, randomized, controlled clinical trial. Based on data from previous research, the statistical power was > 90% (two-sided, alpha = 0.05) with 8 patients per study arm. Assuming an attrition rate of 20% over the course of the study, we enrolled 20 patients in total (10 per group).

### Patients

Patient recruitment began in March 2014. Eligible participants met the following inclusion criteria: 18 to 65 years old without a gender preference; an indication of deterioration in expanded skin texture, such as thin/papery skin, angiotelectasia, and dermis striatum that showed no improvement after suspending expansion for 2 weeks; and a requirement for further expansion to achieve reconstruction. All patients were assessed by two independent plastic surgeons for eligibility.

The exclusion criteria were as follows: (1) a history of severe illnesses, including any cancers, hepatitis, coronary artery disease, arteriosclerosis, diabetes, and obesity (body mass index [BMI] > 30); (2) presence of infection in the expansion area; (3) presence of expanded skin in a haired area; and (4) current smoking or smoking cessation for less than 6 months before enrollment.

### Randomization and masking

Each eligible patient provided written informed consent and was randomly assigned to the SVF or control group in a 1:1 ratio using a computer-generated randomization schedule. The recruiting team randomized the participants, and the conduct team managed the treatments. The evaluating investigator and data collectors were masked to the group allocation. No substantial changes to the study methodology were made after commencement of the trial.

### Intervention protocol

The SVF was isolated as in our preclinical study [20, 21]. Briefly, liposuction was performed under local anesthesia with a wet-swelling solution containing lidocaine and adrenaline, and subcutaneous fat was harvested from the abdomen or posterior inner thigh region depending on the amount of fat that was preserved. The fat was aspirated using an 18-gauge liposuction needle connected to a 20 mL syringe with low pressure. The harvested adipose tissue was washed twice by sterile saline and then centrifuged at 600 rpm for 2 min to remove oil droplets and the swelling solution. Each 20 mL fat sample was incubated with 600 U of collagenase (Shanghai Qiaoyuan Biological Pharmaceutical Co, LTD, Shanghai, China) at 37 °C for 1 h. The sample was then filtered and centrifuged at 1500 rpm for 5 min to obtain the pelleted SVF. The liquid above the red SVF cell-pellet (Fig. 2a) was discarded and the pellet was suspended with saline twice. The cell concentration was adjusted to 1*10^6^ cells/mL in 1 mL syringes for treatment. The skin was pinched to facilitate the intradermal injection of the SVF suspension via a 30-G needle with approximately 0.1 mL injected at a 1 cm interval (1 × 10^5^ cells/cm^2^) (Fig. 2b). The control group was injected with an equal volume of sterile saline in the same manner.

The expander was inflated every 3 days, and a pressure meter was used to monitor the inflation pressure. Inflation was discontinued when the inflation pressure reached 100 mmHg. This procedure was repeated until sufficient tissue was obtained or the maximal regenerative assessment was achieved to perform skin flap transfer surgery.

### Assessment and endpoint

Outcomes were assessed at baseline (immediately before treatment) and at 4, 8, and 12 weeks after the first treatment. The primary endpoint was the change in skin thickness at 12 weeks posttreatment. The secondary endpoints were the changes in the skin thickness including the epidermal and dermal thicknesses at 4 weeks and 8 weeks posttreatment; the expansion index (EI) and the subjective scores of skin texture at 12 weeks posttreatment. Safety assessments were administered at 2 years.

#### Expanded skin thickness

Skin thickness was measured at eight evenly spaced measurement points along the longitudinal axis using a duplex ultrasonic scanner [33]. In ultrasound images, skin appears as a well-defined, linear echogenic band between the air-epidermal band and the low echo-level hypodermal band. Epidermal thickness was defined as the distance between the most superficial, clearly visual high echo-level band, and the dermal thickness was defined as the distance between the moderate-echogenic band underneath the epidermal band.

#### Expansion index

The inflated volumes at each visit were recorded and summed. Due to the maximum capacity of the patient’s expanders, we used the expansion index (EI) to measure the efficiency of the inflation process; the EI was described as the ratio of the total inflated volume (mL) to the designed volume expander (mL).

#### Subjective scores of expanded skin texture

The investigator-assessed subjective scores of expanded skin texture were as follows: 3 indicated that the skin texture was significantly improved and that the optimal desired result was obtained; 2 indicated that the skin texture was significantly improved, but further treatments were required; 1 indicated that the skin texture was slightly improved; 0 indicated that the skin texture was the same as that before treatment; and − 1 indicated that the skin texture was worse than that before treatment. The same blinded investigator assessed all scores.

#### Histological examination

Expanded skin specimens were harvested during flap transfer surgery. Samples were fixed overnight in 4% paraformaldehyde and embedded in paraffin. Samples were sectioned at a thickness of 5 μm, and the slides were deparaffinized and rehydrated. Hematoxylin and eosin (HE) staining (Solarbio, G1120) and Masson’s trichrome (MT) staining (Solarbio, G1340) were performed following standard protocols.

For immunohistochemical staining, dewaxing and rehydrating sections were consumed in a peroxidase-blocking solution, followed by heat-induced epitope retrieval. The sections were incubated at 4 °C overnight with the rabbit anti-human CD31 (Boster, BA2966, 1:100) and mouse anti-human and proliferating cell nuclear antigen (PCNA) (Boster, BM0104, 1:100) antibodies. After overnight incubation, the sections were washed twice with PBS and then incubated with secondary antibodies at 37 °C for 1 h. Sections were detected using horseradish peroxidase and visualized with 3,3′-diaminobenzidine (DAB) staining and counterstaining with Mayer’s hematoxylin for 5 min.

The histologic differences in HE staining in both groups were observed, and collagen synthesis was evaluated using the collagen volume fraction (CVF) based on MT staining. Immunohistochemistry with PCNA was used to evaluate the role of the SVF in promoting skin cell proliferation. Vascular endothelial cells were identified as the surrounding vessels using an anti-CD31 antibody to evaluate angiogenesis in the skin.

The number of positive cells was calculated using a laser confocal microscope (Leica, Wetzlar, Germany) in 5 high-power random fields (HPFs), and the histological analysis was performed objectively by independent investigators.

#### Safety assessments

During the 2-year follow-up period, the following factors were evaluated to identify adverse reactions: redness, swelling, pain, infectious symptoms and symptoms related to the presence of a subcutaneous protuberance, mass, induration, or hyperplasia.

### Study oversight

This study was granted ethical approval from the Institutional Ethics Committee at the Shanghai Ninth People’s Hospital in accordance with the principles of the Declaration of Helsinki. All treatments and follow-up visits were performed at the Department of Plastic and Reconstructive Surgery of Shanghai Ninth People’s Hospital, Shanghai Jiaotong University School of Medicine from March 2014 to December 2018. This trial was registered with the Chinese Clinical Trial Registry, ChiCTR2000039317 (registered 23 October 2020— retrospectively registered, http://www.chictr.org.cn/showproj.aspx?proj=62738).

### Statistical analysis

GraphPad Prism 7 (GraphPad Software Inc., San Diego, CA, USA) was used for the statistical analysis. Continuous data were expressed as the mean ± standard deviation (SD) with a 95% confidence interval (CI), and categorical data were represented as counts. The between-group differences at each visit were performed using two-way repeated-measures ANOVA followed by Sidak’s multiple comparison tests, and within-group differences of each follow-up visit compared to baseline were analyzed using the paired *t* test. The EI increment between groups was assessed using the unpaired *t* test. A *p* value < 0.05 indicated that the difference was statistically significant (*p < 0.05, **p < 0.01).

## Results

### Participant characteristics

**Fig. 1:**
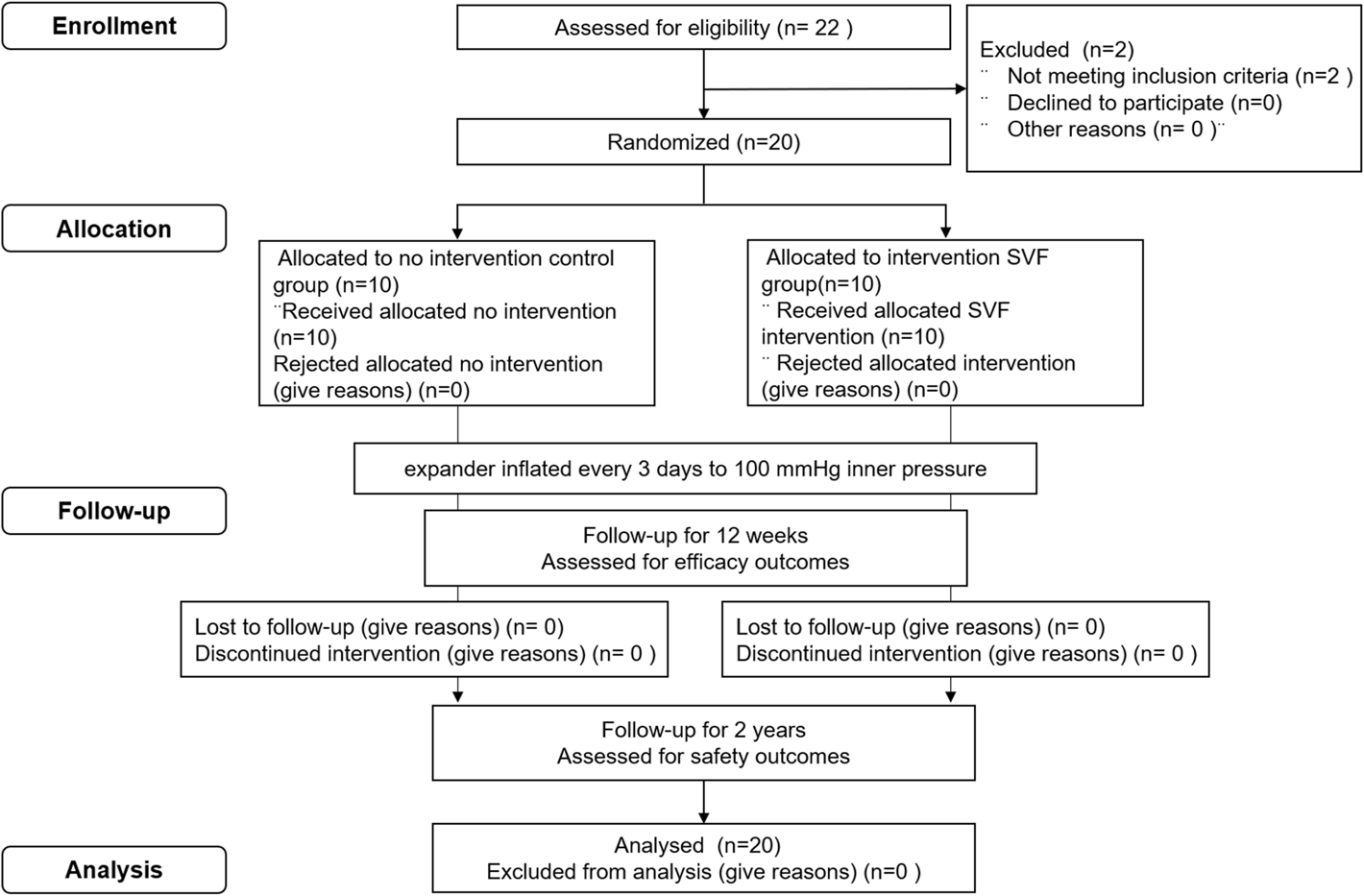
CONSORT flow diagram of participant recruitment

In total, 22 patients were screened from March 2014 to December 2016. Two patients were excluded from the study, 10 patients were randomly assigned to the treatment group, and 10 were randomly assigned to the placebo group (Fig. 1). All patients completed the follow-up visits; none were lost to follow-up. In total, 11 male and 9 female subjects were included with average ages of 24.5 ± 4.4 years in the control group and 26.8 ± 5.9 years in the SVF group. The average duration of the expander implantation was 13.5 ± 5.4 months in the control group and 12.7 ± 5.7 months in the SVF group, and the EI index was 2.08 ± 0.68 in the control group and 2.39 ± 0.59 in the SVF group. The baseline demographic and disease characteristics showed no difference between the groups, as shown in Table 1.

**Table 1:** Patient demographic and baseline characteristics

	Control group (***n*** = 10)	SVF group (***n*** = 10)	***P*** value
Age	24.5 ± 4.4	26.8 ± 5.9	0.336
Gender			
Male	6	5	–
Female	4	5	–
Body mass index (BMI)	22.6 ± 3.5	21.1 ± 3.1	0.307
Expander implantation site			
Cervical	5	2	–
Chest	1	4	–
Dorsal	2	3	–
Facial	2	1	–
Expander implantation time (month)	13.5 ± 5.4	12.7 ± 5.7	0.751
Expansion index (EI)	2.08 ± 0.68	2.39 ± 0.59	0.993
Subcutaneous fat donor area			
Abdomen	4	5	–
Hip	6	5	–
Number of transplanted cells(10^6^)	–	2.96 ± 1.17	–

### Primary outcome

#### Expanded skin thickness at 12 weeks

**Fig. 2:**
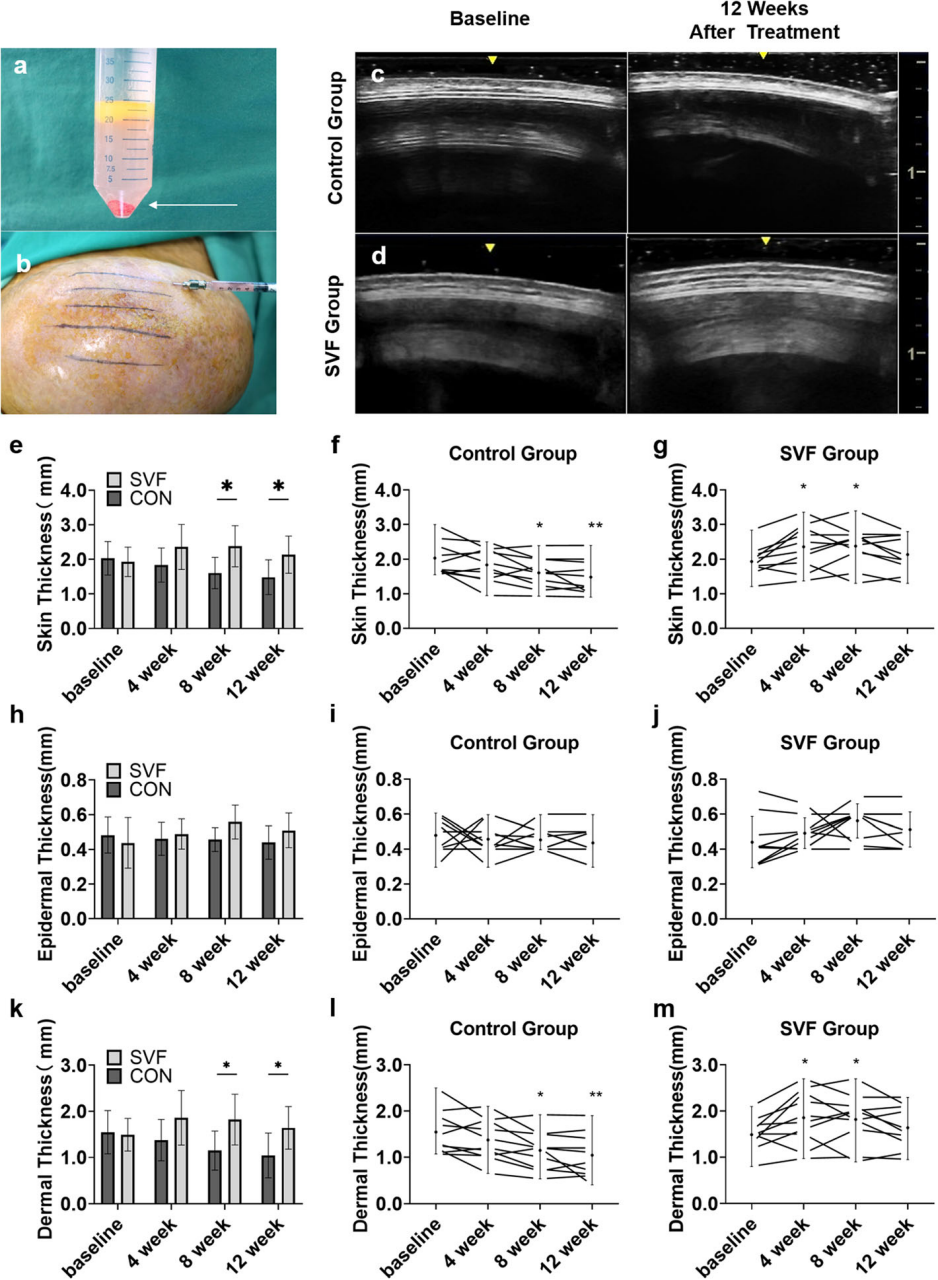
The SVF injection and the changes in skin thickness. Through liposuction and enzyme digestion, the red SVF cell-pellet (shown in arrow) was harvested (**a**). SVF was subcutaneously injected onto the expanded skin area (**b**). An ultrasound was used to assess the skin thickness (results from the control group (**c**) and SVF group (**d**) at baseline and 12 weeks). The SVF group had a significant increase in the skin thickness compared to the control group at 8 to 12 weeks (**e**). Compared to baseline, the skin thickness in the SVF group increased significantly at 4 to 8 weeks and was maintained after 12 weeks, whereas the control group decreased after 8 weeks (**f**, **g**). A similar downward trend was found for the dermal thickness (**l**, **m**), whereas no differences in the epidermal thickness were noted between both groups (**i**, **j**).* *p*<0.05; ** *p*<0.01

At baseline, patients from both groups had similar skin thicknesses (control group 2.03 (0.48) mm vs. SVF group 1.93 (0.38) mm, *p* = 0.957). The SVF group had significantly thicker skin (2.14 (0.54) mm) than the control group had (1.48 (0.50) mm) at 12 weeks posttreatment (mean difference 0.65 [95% CI − 1.30 to − 0.01]; *p* = 0.046). At 12 weeks, the skin thickness of the SVF group was still greater than that at baseline, whereas that of the control group was significantly decreased (Fig. 2) (Table 2).

**Table 2:** Skin thickness and EI parameter at baseline (V1), and after 4 (V2), 8 (V3), 12 (V4) weeks

Group	Control group (***n*** = 10)				SVF group (***n*** = 10)					
	Mean	± SD	***P*** value (visit V.S. V1)		Mean	± SD	***P*** value (visit V.S. V1)		***P*** value CON V.S. SVF	
Skin thickness (mm)										
V1	2.03	0.48	–	–	1.91	0.42	–	–	0.981	ns
V2	1.84	0.49	0.150	ns	2.36	0.65	0.010	*	0.214	ns
V3	1.61	0.45	0.037	*	2.38	0.59	0.026	*	0.018	*
V4	1.48	0.50	0.008	**	2.14	0.54	0.144	ns	0.046	*
Epidermal thickness (mm)										
V1	0.48	0.10	–	–	0.44	0.15	–	–	0.900	ns
V2	0.46	0.09	0.710	ns	0.49	0.09	0.095	ns	0.936	ns
V3	0.45	0.07	0.534	ns	0.56	0.10	0.086	ns	0.062	ns
V4	0.44	0.10	0.315	ns	0.51	0.10	0.251	ns	0.421	ns
Dermal thickness (mm)										
V1	1.55	0.47	–	–	1.49	0.35	–	–	0.997	ns
V2	1.37	0.45	0.135	ns	1.86	0.59	0.029	*	0.199	ns
V3	1.15	0.42	0.031	*	1.82	0.55	0.048	*	0.028	*
V4	1.04	0.49	0.007	**	1.64	0.46	0.189	ns	0.045	*
EI Index										
V1	2.39	0.57	–	–	2.39	0.47	–	–	1.000	ns
V4	2.83	0.55	0.000	**	3.06	0.79	0.002	**	0.209	ns
Increment EI										
V4-V1	0.44	0.25	–	–	0.94	0.70	–	–	0.047	*

* *p*<0.05, ** *p*<0.01, ns *p*>0.05

### Secondary outcome

#### Expanded skin thickness at 4 and 8 weeks

The skin thickness in the SVF group significantly increased at 4 weeks to 2.36 (0.65) mm (mean difference 0.49 [95% CI − 0.80 to − 0.06]; *p* = 0.010), and the increase was maintained at 8 weeks (2.38 (0.59) mm (mean difference 0.45 [95% CI − 0.92 to 0.02]; *p* = 0.026)). In the control group, the skin thickness deteriorated after 4 weeks.

The main contributor to skin thickness changes was the dermis. The epidermal thickness was similar in the two groups. Compared to the baseline, the dermis in the SVF group was significantly thickened at 4 weeks (1.86 (0.59) mm) (mean difference 0.37 [95% CI − 0.77 to 0.03]; *p* = 0.029) and 8 weeks (1.82 (0.55) mm) (mean difference 0.33 [95% CI − 0.73 to 0.08]; *p* = 0.048). In contrast, the dermal thickness in the control group decreased continuously (Fig. 2) (Table 2). These findings indicate that SVF treatment could promote skin regeneration by increasing dermal thickness to prevent the expanded skin from becoming papery. In contrast, the control group exhibited thinning of the skin after inflation.

#### Expansion index

**Fig. 3:**
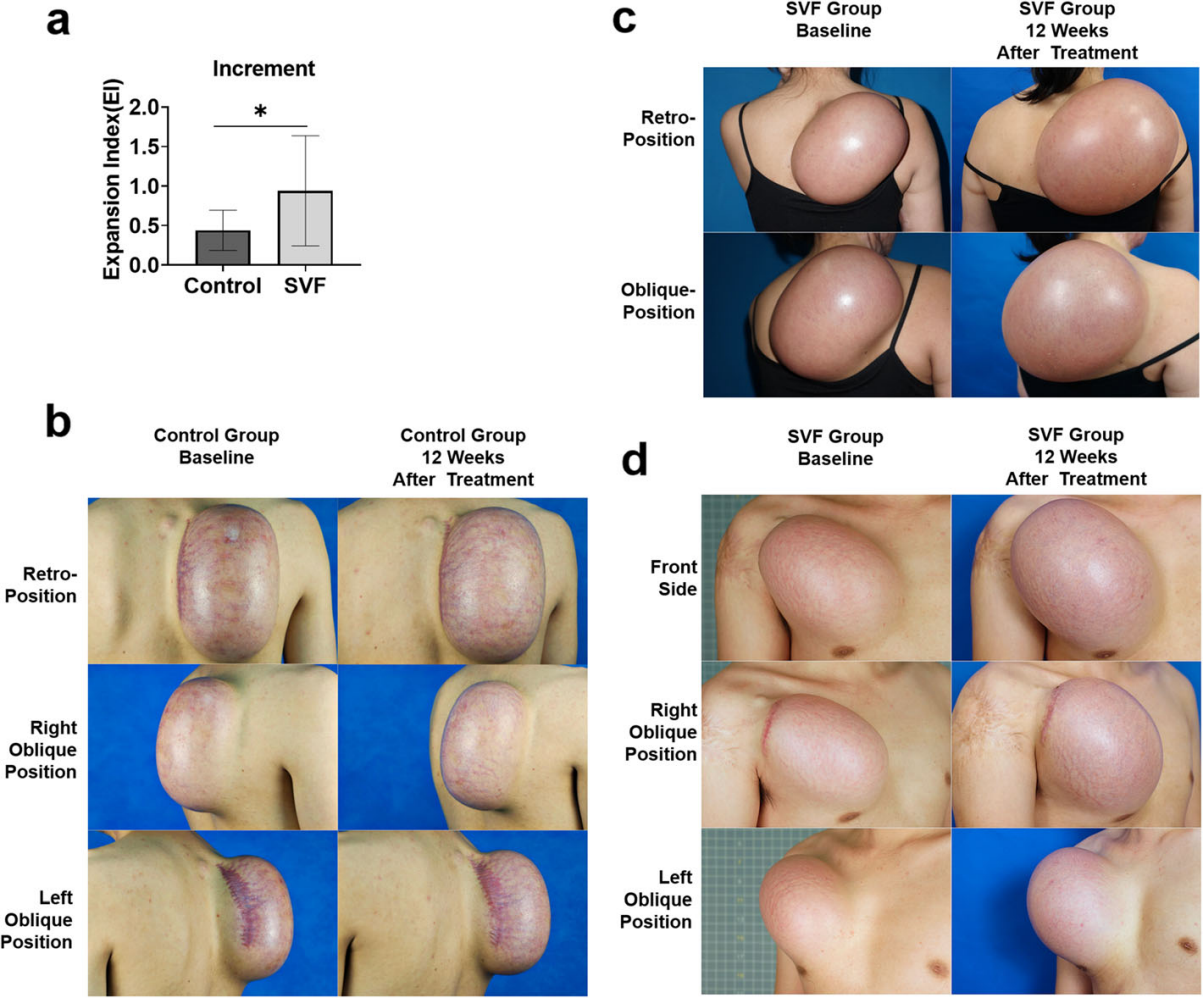
Changes in the EI. The EI was significantly increased in the SVF group (**a**). Patients in the SVF group gained significantly more skin surface area (**c**, **d**) compared with control group (**b**) at 12 weeks of treatment. *p<0.05

The EI increase from baseline to 12 weeks was significantly increased in the SVF group (0.940 (0.698) versus the control group (0.440 (0.255)) (mean difference 0.50 [95% CI − 0.00 to 0.99]; *p* = 0.047) (Fig. 3). The results indicate that the SVF group exhibited increased inflation volumes and larger skin areas at the end of follow-up.

#### Skin texture evaluation

**Fig. 4:**
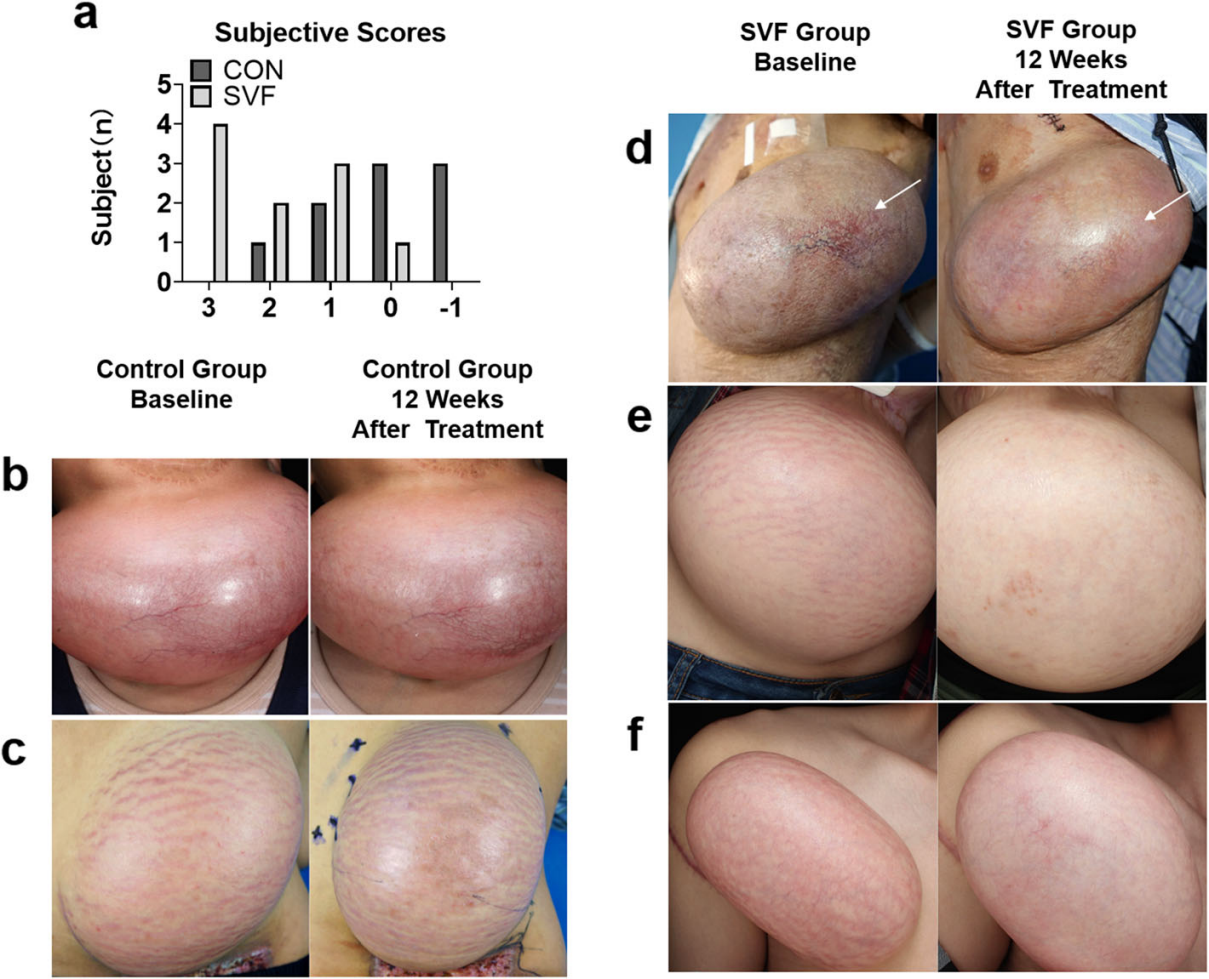
Evaluation of skin texture. Patients in the SVF group had higher scores when their expanded skin texture was assessed compared to patients in the control group at 12 weeks (**a**). Compared to baseline, patients in the SVF group showed significantly improved skin textures (**d**–**f**) at 12 weeks posttreatment but those in the control group had no improvement (**b**, **c**). The skin texture of patients in the control group was deteriorated after 12 weeks posttreatment. This deterioration was noted by increased telangiectasia (**b**) and a deepening of the stretch striae (**c**). **d** Prior to study enrollment, the patient had papery skin accompanied by telangiectasia and the development of an embolism (shown by the arrow). The patient was facing expansion failure. After SVF treatment, his skin texture improved, and its thickness increased. The telangiectasia area was diminished (shown by the arrow). The patient gained further expansion until reaching the need for reconstruction. **e** The patient had stretch marks throughout the expanded skin at baseline. After 12 weeks of SVF treatment, the stretch striae had disappeared, and skin growth improved. **f** Before study enrollment, the patient’s expanded skin appeared papery with stretch striae, and expansion could not be continued. After 12 weeks of SVF treatment, the skin had increased in thickness, and the stretch marks diminished

The blinded investigator subjectively evaluated the skin texture scores at 12 weeks. The SVF group showed significant improvement in skin texture compared to the control group given that the number of patients scored as “significantly improved” and “improved” was greater in the SVF group (grade 3: 4 [40%] and grade 2: 2 [20%] vs. grade 3: 0 [0%] and grade 2: 1 [10%]) (Fig. 4a). The improved skin textures in the SVF group at their 12-week assessment are highlighted in Fig. 4. Fewer patients in the treatment group complained of thin, papery/transparent skin compared to the control group during the follow-up.

#### Histological examination

**Fig. 5:**
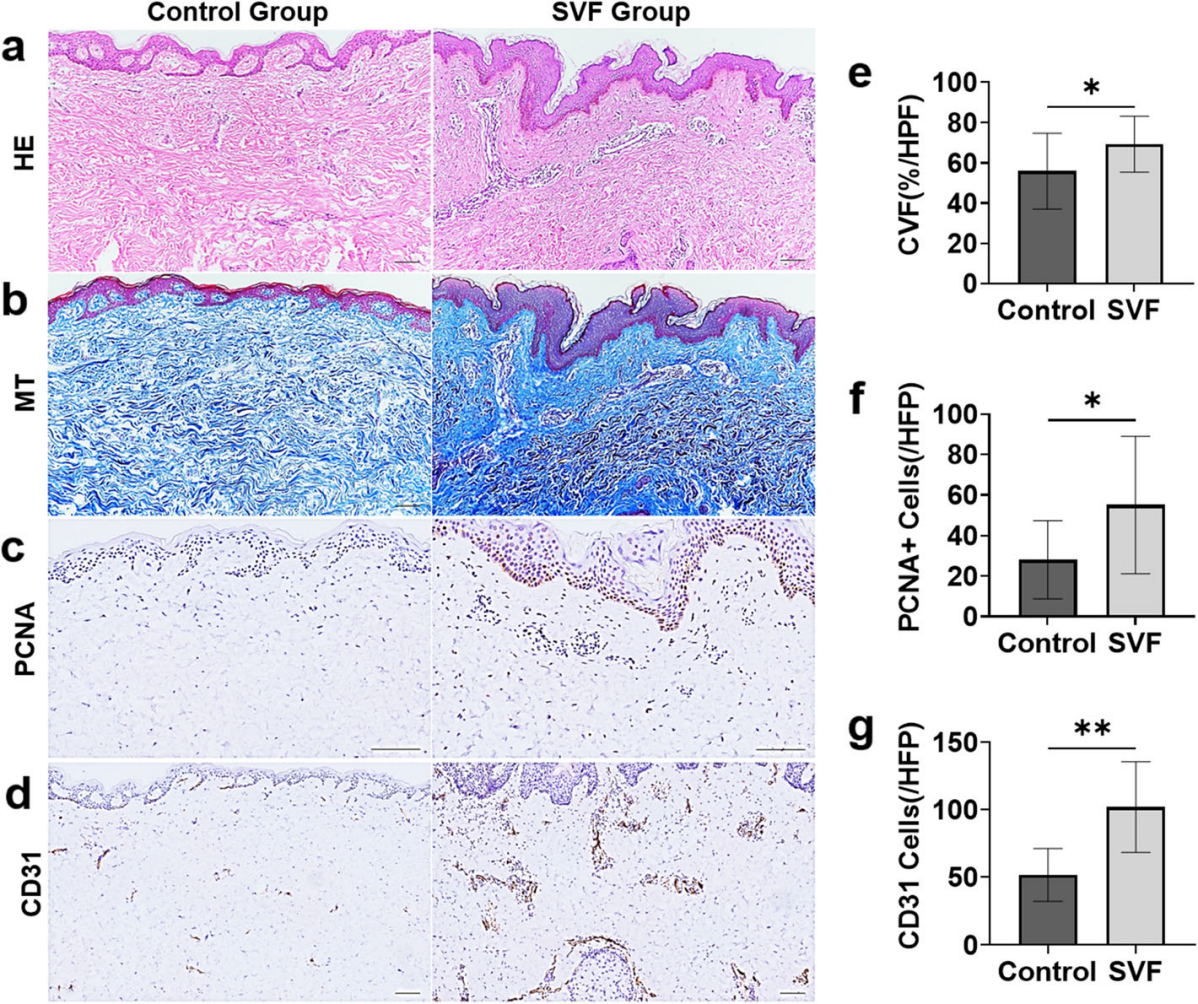
Histology and immunohistochemical staining of the expanded skin. HE staining showed that the epidermal layer in the SVF group was thicker than that in the control group (**a**) with the presence of an increasing rete subpapillary in the papillary layer. Masson’s trichrome staining showed that the ECM volume in the SVF group was significantly increased as the collagen fibers showed an organized distribution, whereas the collagen fibers in the control group (**b**) were loosely organized. PCNA + proliferating cells and CD31+ stained vessels were significantly increased in the SVF group compared to the control group (**c**, **d**). The results of the statistical analysis showed more proliferating cells (**e**) and blood vessels (**f**) in the SVF-treated skin as the volume of ECM increased (**g**). Scale bar in **a**–**d**, 100 μm. *p<0.05, **p<0.01

HE staining results show that the epidermal layer in the SVF group was thicker than that in the control group with the presence of an increasing rete subpapillary in the papillary layer (Fig. 5a). The papillary dermis was thickened, and the collagen fibers were increased with an organized distribution in the SVF group. However, the collagen fibers in the control group were loosely organized (Fig. 5b). MT staining showed that the CVF in the SVF group was 54.1 (19.9)%/HPF, which is significantly greater than that in the control group (71.1(14.7) %/HPF, *p* = 0.049) (Fig. 5e).

Compared with the control group, the number of PCNA + proliferating cells was significantly increased in the SVF group (55.1 (33.9)/HPF versus 28.1(19.3)/HPF in the control group, *p* = 0.042) (Fig. 5f). In the SVF group, proliferating cells presented not only in the basal layer of the epidermis but also in the papillary layer of the dermis (Fig. 5c). Increased vascular density throughout the dermis was observed in the SVF group (Fig. 5d) (26.6(12.2)/HPF versus 17.2(6.5)/HPF in the control group, *p* = 0.045) (Fig. 5g), indicating improved vascularization after SVF injection.

#### Safety

After the injection, 12 participants (60%) had temporary ecchymosis in the injection area; however, the symptoms disappeared within a week without sequelae. None of the participants had infectious or severe adverse events during the 24-week follow-up period. During the 2-year follow-up, subcutaneous protrusion, mass, induration, or hyperplasia were not found.

## Discussion

Stimulating skin with mechanical stretching induces a cellular response and results in ECM synthesis, vascularization, and the proliferation of fibroblasts and epidermis cells [34–36]. Skin expansion is achieved through the application of mechanical stretch-induced skin regeneration in reconstructive surgery and is used to obtain regenerated tissue [2]. However, the expansion of skin is limited by the intrinsic capacity of skin to regenerate [4, 6]. Numerous studies have explored methods to promote skin regeneration, including the use of prostaglandin E2, papaverine, cytochalasin, and dimethyl sulfoxide (DMSO) for expediting skin expansion, but no study has established sufficient clinical efficacy [37–39]. The mechanisms involved in regenerative exhaustion in skin remain debated, and recent findings suggest that stem cell deregulation or insufficiency is involved [40, 41]. Therefore, we need to explore new methods to promote tissue expansion by enhancing skin regeneration.

In our previous research [19–22, 42], we found that combining stem-cell therapy with mechanical stretch-induced skin regeneration could overcome the limitation of stretch-induced regeneration. In animal studies, we demonstrated that stem cells from both bone marrow and adipose tissue could improve skin regeneration. The efficacy of bone marrow stem cells in rescuing thinning expanded skin was demonstrated by our previous randomized clinical trial [33]. However, harvesting mononuclear cells (MNC) is a painful procedure that produces only a limited number of cells. In contrast, large amounts of SVF can be obtained through a minimally invasive liposuction procedure, suggesting that SVF cells are a more appropriate source for clinical cell-based therapies. Hence, in this study, we discussed the clinical efficacy and safety of SVF in stimulating mechanical stretch-induced tissue regeneration.

The results from this study indicate that intradermal transplantation of SVF aids in sustainable regeneration of expanded skin that shows signs of regenerative exhaustion. The SVF group showed statistically significant improvement in primary and secondary outcomes compared with both the control group and the baseline. After SVF treatment, the thickness of the expanded skin significantly increased at 4 and 8 weeks after treatment and was maintained at 12 weeks. In the control group, the skin thickness showed a continuous decrease during the follow-up. Approximately 60% of the cases in the SVF group were evaluated as having an improved texture, whereas only 10% of the control group had better skin texture compared with baseline. Our results show that intradermal injection of SVF improved the thinning and deterioration of expanded skin and aided in continuous skin regeneration.

Adipose-derived stem cells (ADSCs) are the primary source of adult stem cells with regenerative potential, and these cells are isolated from lipoaspirates by enzymatic digestion/mechanical centrifugation [43, 44]. The lipoaspirates contain a combination of ADSCs, endothelial progenitor cells (EPCs), endothelial cells (ECs), macrophages, smooth muscle cells, lymphocytes, pericytes, and preadipocytes, and this mixture is referred to as the stromal vascular component (SVF). SVF is a research hotspot, even garnering more interest than ADSCs at present, given that it participates in tissue regeneration via multiple mechanisms [23, 25] and is involved in anti-apoptotic, immunomodulatory, anti-inflammatory, and angiogenesis actions [45]. EPCs and ADSCs may contribute to these actions. EPCs serve as endothelial progenitors, endothelial cells, and pericytes, which contributed to neovascularization and vessel remodeling [46]. ADSCs can secrete growth factors and chemotactic factors, such as vascular endothelial growth factor (VEGF), fibroblast growth factor- 2(FGF-2), hepatocyte growth factor (HGF), and interleukin 6 (IL-6), which are involved in regulating immune, anti-inflammatory, and angiogenesis responses [26, 27, 47]. The coculture of ADSCs and ECs increased the production of ECM, which stabilized the newly formed vessels [48]. Studies have shown that SVF treatment enhances the density of capillaries, expedites the healing process in the wounds of mice, improves the hair growth of skin appendages, and promotes vascularization of ischemic limbs in the murine model [49–52]. In our study, the histological results of our trial show that more blood vessels were observed in tissue from the SVF group. Additionally, the numbers of proliferating basal cells and fibroblasts increased as the volume of ECM increased.

SVF treatment delivers a better therapeutic effect without increasing adverse events. No subcutaneous protrusions, masses, induration, or hyperplasia was found in the SVF group. Although the ecchymosis spontaneously resolved, it decreased the participants’ satisfaction and compliance.

As noted in our previous study [33], MNC treatment increased the skin thickness after 4 weeks, but the increase consistently declined after 8 weeks following inflation. Compared to SVF treatment, the therapeutic effect of the cellular component of SVF offers more lasting effects for mechanical stretch-induced skin regeneration.

There are still some limitations in our study. Further randomized controlled trials with larger sample sizes and longer follow-up durations are warranted. The potential immunogenicity and tumorigenicity of the SVF treatment must be investigated before clinical use. We need to further explore the cellular mechanism of SVF transplantation in promoting skin regeneration under conditions of mechanical stretching. Although the histological results showed more proliferating cells and angiogenesis in the SVF treatment group, the transplanted stem cells could not be tracked in vivo.

## Conclusion

This clinical trial demonstrated that the application of SVF is reliable and efficient in overcoming the limitations of mechanical stretch-induced skin regeneration. The SVF group showed better outcomes in expediting the potency of skin expansion, increasing the dermal thickness, and ensuring a sufficient inflation volume compared with the control group. We believe that the synergistic combination of stem cells and mechanical stretching stimulation will promote the further development of tissue regeneration in vivo and help to achieve more satisfactory reconstruction outcomes.

## Data Availability

The datasets used and/or analyzed during the current study are available from the corresponding author on reasonable request.

## Abbreviations

BMI: Body mass index
CI: Confidence interval
CVF: Collagen volume fraction
DMSO: Dimethyl sulfoxide
ECM: Extracellular matrix
EI: Expansion index
HE: Hematoxylin and eosin
HPF: High-power field
MT: Masson’s trichrome
MNC: Mononuclear cells
PCNA: Proliferating cell nuclear antigen
SD: Standard deviation
SVF: Stromal vascular fraction
